# Utilising Co-Axial Electrospinning as a Taste-Masking Technology for Paediatric Drug Delivery

**DOI:** 10.3390/pharmaceutics13101665

**Published:** 2021-10-12

**Authors:** Hend E. Abdelhakim, Alastair Coupe, Catherine Tuleu, Mohan Edirisinghe, Duncan Q. M. Craig

**Affiliations:** 1 Department of Pharmaceutics, UCL School of Pharmacy, 29-39 Brunswick Square, London WC1N 1AX, UK; hend.abdelhakim@ucl.ac.uk (H.E.A.); C.tuleu@ucl.ac.uk (C.T.); 2 Pfizer Limited, Global R&D, Discovery Park, Ramsgate Road, Sandwich, Kent CT13 9ND, UK; alastair.coupe@pfizer.com; 3 UCL Department of Mechanical Engineering, Faculty of Engineering Sciences, University College London, London WC1E 7JE, UK; m.edirisinghe@ucl.ac.uk

**Keywords:** co-axial electrospinning, taste-masking, Eudragit E PO, Kollicoat Smartseal, E-tongue, chlorpheniramine maleate, taste-assessment

## Abstract

The present study describes the use of two taste-masking polymers to fabricate a formulation of chlorpheniramine maleate for paediatric administration. Co-axial electrospinning was utilized to create layered nanofibres; the two polymers, Eudragit^®^ E PO and Kollicoat^®^ Smartseal, were alternated between the core and the shell of the system in order to identify the optimum taste-masked formulation. The drug was loaded in the core on all occasions. It was found that the formulation with Kollicoat^®^ Smartseal in the core with the drug, and Eudragit^®^ E PO in the shell showed the most effective taste-masking compared to the other formulations. These fibres were in the nano-range and had smooth morphology as verified by scanning electron microscopy. Solid-state characterization and thermal analysis confirmed that amorphous solid dispersions were formed upon electrospinning. The Insent E-tongue was used to assess the taste-masking efficiency of the samples, and it was found that this formulation was undetectable by the bitter sensor, indicating successful taste-masking compared to the raw version of the drug. The E-tongue also confirmed the drug’s bitterness threshold as compared to quinine HCl dihydrate, a parameter that is useful for formulation design and taste-masking planning.

## 1. Introduction

Poor taste of medicines is one of the main adherence challenges for the paediatric population, hence it is necessary to design specific dosage forms suitable for administration to children [1]. Nevertheless, age appropriate paediatric medicines have traditionally been in comparatively low abundance, a fact that led to the introduction of the EU 2007 Paediatric Regulation [2]. This legislation stipulates that, for any new drug, the manufacturer is required to develop an accompanying plan on how the medicine can be practically administered to children, formally known as a Paediatric Investigation Plan, or PIP. Waivers are granted to medicines intended for use in diseases that occur only in adults. As part of the Paediatric Regulation, Paediatric Use Marketing Authorizations, or PUMAs, were also introduced [3]. These incentivized manufacturers to re-formulate existing drugs on the market into age-appropriate formulations through improving various acceptability attributes, most notably taste. This therefore created a significant window of opportunity for formulators to re-design bitter drugs using innovative techniques to produce palatable age appropriate formulations that may be eligible for PUMA status [4].

Dispersible oral films have a well-recognised potential for use in children due to their numerous advantages over conventional dosage forms, including favourable mouthfeel, improved swallowability, and lack of the (absolute) necessity to take with water [5]. Electrospinning is a manufacturing method that can produce fibre mats which may in turn be processed into oral films [6]. While electrospinning has been previously explored as a taste-masking platform technology [7,8,9,10,11], considerable further work is required to develop formulation and production strategies so as to ensure that the advantages of rapid dispersion and taste-masking may be successfully combined. The potential for such systems is, however, considerable, especially as one of the main perceived challenges of electrospinning, that of small scale production, has recently been overcome via the availability of new scaling technologies [12]. Therefore, the possibility of producing highly tailored electrospun systems that may be effective at masking taste (via appropriate choices of polymers), as well as being aesthetically acceptable to the child, merits further exploration.

In this study, co-axial electrospinning was utilized to produce a taste-masked pre-formulation system with the intention of using such a material to produce a palatable oral film to be used in children. In a previous study [11], Quality by Design (QbD) principles including Design of Experiment (DoE) were applied to optimize the electrospinning of Eudragit^®^ E PO (E-EPO), a pH sensitive and moisture protective taste-masking polymer. This involved the incorporation of a model bitter drug, chlorpheniramine maleate (CPM), a bitter antihistamine commonly used in children. A DoE approach was utilised to identify the optimum electrospinning process and solution parameters to produce bead-free nanofibres. For example, it was found that the E-EPO’s chain entanglement concentration was between 30 and 35% *w/v* (optimizing film formation properties) and the optimum solvent to use in order to optimize the parameters mentioned was ethanol.

**Figure 1:**
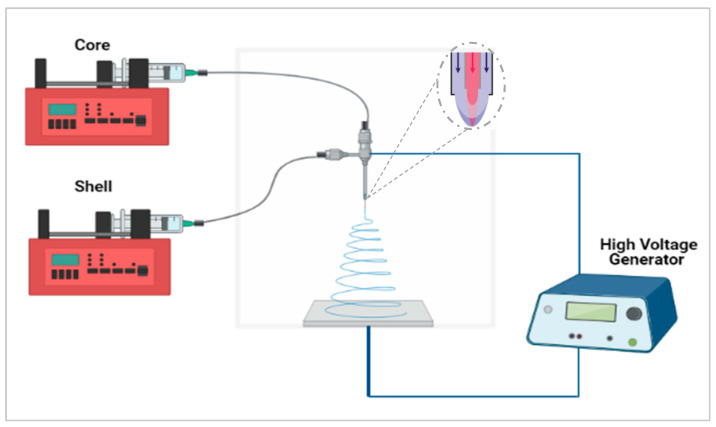
Co-axial electrospinning setup schematic. Created with https://biorender.com/; accessed on 1 June 2021.

In this study, the aim was to maximize the taste-masking of the formulation through the use of another taste-masking polymer, Kollicoat^®^ Smartseal (KCT), to produce core–shell fibres using co-axial electrospinning. This approach, illustrated in Figure 1, incorporates two needles embedded within each other, creating a core and shell solution which solidify on extrusion to form a bilayered system [13,14]. A variety of materials may be used to produce fibres or mats with varying functions such as rapid or controlled release [15] or, as in this case, taste-masking. It has been reported that both solutions need not to be electrospinnable, especially the core solution, as the shell solution can ensure the viability of the electrospinning process [16]. There are therefore a variety of options for the design and development of the fibres; in the context of taste-masking this flexibility may be exploited to allow both effective taste-masking and optimal handling properties.

As a continuation of the authors’ earlier research, the model bitter drug used was CPM. In addition to using E-EPO, KCT was also used to taste-mask the bitter drug. KCT is a moisture and pH sensitive polymer (insoluble at neutral pH but readily soluble at pH < 5) that conceals bitter tastes and creates a moisture barrier around the formulation [17]. As there are no previous reports in the literature of KCT being electrospun, it was initially explored whether KCT can indeed be electrospun, and if so, what the optimum spinning conditions may be. In addition, we examine whether co-axial electrospinning using these two polymers is more effective in masking taste than using either of the polymers alone. Taste-assessment of pharmaceutical dosage forms is very important to ensure a medicine will be deemed palatable by the target population [18]. Palatability is defined as the overall acceptability of a medicines organoleptic properties such as its appearance, smell, taste, mouthfeel, and after-taste [19]. Human taste panels are considered to be the gold standard in bitter drug taste-assessment; however, these can be difficult to run due to ethical and safety concerns [20]. In-vitro taste-assessment methods are more accessible and comprise two main options, dissolution or the use of an electronic tasting system or E-tongue [21].

In this study, an E-tongue was used to taste-assess the electrospun formulations. The E-tongue ‘tastes’ substances based on potentiometric measurement principles. The basic system consists of a working lipid taste sensor and a reference taste sensor; when a potential difference is generated between the two via immersion in a test solution, this reading may be correlated to bitterness or indeed a number of taste sensations depending on the design and composition of the sensor [22,23].

It is reasonable to suggest that a co-axial approach may be advantageous compared to a single layered system, as the former would theoretically not only provide an additional covered layer to the drug but would also negate surface release effects if the drug is incorporated into the core of the fibres. The overarching aim for the study was to therefore examine how the architecture of the co-axial systems may be related to taste-masking, as verified by the E-tongue, and to establish whether using a core–shell approach may indeed be advantageous compared to single layered systems.

## 2. Materials and Methods

### 2.1. Materials

E-EPO, described as basic butylated methacrylate copolymer in the European Pharmacopoeia, is a white powder with an average molar mass of approximately 47,000 g/mol [24] and was kindly donated by Evonik (Darmstadt, Germany). KCT 30D, methyl methacrylate and diethylaminoethyl methacrylate (7:3) copolymer was kindly donated by BASF (Ludwigshafen, Germany) [17]. CPM was purchased from Cambridge Bioscience (Cambridge, UK). Rhodamine B was purchased from Sigma-Aldrich (Dorset, UK). Acetone and ethanol were the solvents used and were obtained from Sigma-Aldrich (Dorset, UK). Tartaric acid, potassium chloride, and potassium hydroxide were obtained from Sigma-Aldrich (Dorset, UK). Hydrochloric acid was obtained from Fisher Chemicals (Loughborough, UK). All substances were used as received.

### 2.2. Preparation of Precursor Solutions

E-EPO solutions were prepared by dissolving 30–35% *w/v* E-EPO in absolute ethanol. The mixture was then magnetically stirred for approximately two hours. The solution was allowed to rest for a day at ambient conditions before electrospinning. Drug-loaded E-EPO solutions were prepared by adding CPM directly to the polymer solution at a concentration of 3.5% *w/v*.

KCT 30D, an aqueous solution, is miscible with ethanol at a ratio of 1:3. As it comes as a 30% *w/v* solution, after dilution with ethanol a 7.5% *v/v* polymer solution is formed. For drug-loaded systems, CPM was directly added to the polymer solution at a concentration of 3.5% *w/v*.

Rhodamine B (0.01% *w/v*) was added to the core solutions for viewing with fluorescence microscopy.

### 2.3. Electrospinning

A Spraybase electrospinning instrument (Spraybase, Dublin, Ireland) was used to manufacture the fibres. In the electrospinning process, the prepared solution was drawn in a 5 mL Terumo syringe (Surrey, UK), attached to a syringe pump. In case of monoaxial electrospinning of KCT, the syringe was connected to a stainless-steel needle, with a diameter of 0.9 mm, via a connector tube. The viscous solution was fed through the needle at flow rates between 0.5 and 2 mL/h. Applied voltages up to 25 kV were applied to the polymer solutions, evaporating the solvent, allowing the solid fibres to deposit on a grounded metal plate collector (14.5 cm × 23 cm). The gap distance between the needle and the collector plate was set between 10 cm and 20 cm. Room temperature (°C) and relative humidity (RH) (%) readings were recorded. The temperature ranged between 21 and 28 °C, and the RH ranged between 26% and 46%.

#### Co-Axial Electrospinning

The co-axial emitter used had a diameter of 0.9 mm for the outside needle and 0.45 mm for the inside needle. A co-axial electrospinning schematic is shown in Figure 1. Inside and outside needles were the same length at 10 mm from the hub. In order to find the optimum formulation for taste-masking CPM, 7.5% (*w/v*) KCT and 35% (*w/v*) E-EPO were alternated between the core and shell solutions. The flow rates used were 0.45 mL/h for both the core and shell solution. The gap distance was set between 15 cm and 17.5 cm and the applied voltage was between 15 kV and 20 kV. 

### 2.4. Fluorescence Microscopy

Core solutions were dyed with Rhodamine B, electrospun and samples collected on a glass microscope slide. Slides were then viewed on an EVOS FL imaging system (Thermo Fisher Scientific, Waltham, MA, USA) under Texas Red light. Images were saved and visually inspected for the core–shell structure.

### 2.5. Scanning Electron Microscopy

A sample of the fibre collected was adhered onto aluminium scanning electron microscopy (SEM) stubs (TAAB Laboratories, Reading, UK) using a carbon-coated double-sided tape. To render them conductive, a thin coating of gold was applied in a Quorum Q150T sputter coater (Quorum Technologies Ltd., East Sussex, UK) in an argon atmosphere. A scanning electron microscope FEI Quanta 200 FEG (FEI Company, Hillsboro, OR, USA) was used to image the fibre morphology. ImageJ 1.46R software (NIH, Bethesda, MD, USA) was used to measure the diameters of the fibres imaged. OriginPro 9.4 (Origin Lab, Northampton, MA, USA) was used to construct the histograms [25].

### 2.6. Transmission Electron Microscopy

Fibres were collected on a copper grid (to allow passage of electrons) during electrospinning. They were then stained with 2% aqueous uranyl acetate solution prior to imaging with a TEM FEI CM120 BioTwin (FEI Company, Hillsboro, OR, USA) at an accelerating voltage of 120 kV.

### 2.7. Differential Scanning Calorimetry

Modulated temperature differential scanning calorimetry (MTDSC) was used to generate thermograms of pure drug, polymer, physical mixture, and electrospun fibres. These data were recorded using a TA Instruments Q2000 calorimeter (TA Instruments, New Castle, DE, USA).

Sample weights ranged from 4 to 8 mg and were sealed in a 40 μL aluminium PerkinElmer standard pan. A pinhole was manually formed in the lids to allow for solvent evaporation. Samples were heated under nitrogen gas (flow rate 50 mL/min) at a heating rate of 2 °C/min ramped up to 150 °C, amplitude ± 0.212 °C, and a period of 40 s. Data analysis were carried out with TA Universal Analysis software, version 4.5A.

### 2.8. UV Spectroscopy and Drug-Loading

Standard solutions of CPM with the concentration range of 5−50 μg/mL were prepared in ethanol. A standard curve (R^2^ = 0.9994) of CPM was plotted using absorbance data recorded using a Jenway 6305 UV−Vis spectrophotometer (Bibby Scientific, Staffordshire, UK). Absorbance was recorded at 262 nm (λmax). Drug-loading was calculated by using the slope = 0.01248 and intercept = 0.04057 of the standard curve.

Film samples weighing approximately 10 mg were measured out and dissolved in 10 mL of ethanol. This was repeated three times. Each sample was tested three times using UV spectroscopy. Therefore, nine samples were tested for each formulation to ensure the reading is both accurate and precise. For each sample, the theoretical drug-load was calculated as follows:

$$Concentration\;of\;each\;Polymer\;\left(\%\frac{w}{v}\right)\times flow\;rate\;\left(\frac{mL}{h}\right)=\;sum\;of\;all\;polymer\;content\;\left(\%\frac{w}{v}\right)\;API\;concentration\;\left(\;3.5\%\;w/v\right)\times flow\;rate\;\left(\frac{mL}{h}\right)=drug\;content\;\left(\%\frac{w}{v}\right)\;Theoretical\;Drug\;loading\;\left(\%\right)=\frac{drug\;content\;\left(\%\frac{w}{v}\right)}{sum\;of\;all\;polymer\;and\;drug\;content\;\left(\%\frac{w}{v}\right)}$$

For example, for a 35% (*w/v*) E-EPO core and 7.5% (*w/v*) KCT shell formulation, the polymer content sum is (0.45 × 35 = 15.75) + (0.45 × 7.5 = 3.375) = 19.125; the drug content is (0.45 × 3.5 = 1.575); and the theoretical drug-load therefore is; 1.575/ (19.125 + 1.575) ∗ 100 = 7.6% (*w/v*). Using this theoretical percentage, the amount of the drug in the samples tested was calculated. The absorbance readings for the samples tested were inserted in the calibration curve to give a concentration measurement. After accounting for dilution factors, a mean number was calculated, which represents the actual drug-loading in the results section. This value was then divided by the theoretical drug amount and multiplied by a 100; this gave rise to a % drug-loading efficiency of the actual drug amount divided by the theoretical drug amount.

As the core and shell solution’s flow rate were set at a 1:1 ratio, the theoretical drug-load of the co-axial system was calculated as follows; for co-axial system 3 which contained 35% (*w/v*) in both the core and shell, the drug-loading was 4.76% (*w/w*). For co-axial system 4 that contained 7.5% (*w/v*) of KCT in both the core and shell solutions, the drug-loading was 18.9% (*w/w*). For co-axial systems 2 and 5 which contained both 35% (*w/v*) E-EPO plus 7.5% (*w/v*) KCT in either the shell or the core, the drug-loading was 7.6% (*w/w*). In all systems the amount of drug actually added was the same at a concentration of 3.5% (*w/v*). 

### 2.9. X-ray Diffraction

Solid state characterization of materials before and after electrospinning were completed using a Rigaku MiniFlex 600 X-ray diffractometer (Rigaku, Tokyo, Japan). Cu Kα radiation was operated at 40 mV and 15 mA. Patterns were recorded over the 2θ range 3°−40° at a scan rate of 3°/min, with an interval of 0.02°. RAW files produced were converted to X-ray diffraction (XRD) data files using PowDLL version 2.51 file converter software. The data were then viewed on X’Pert Data Viewer version 1.2F.

### 2.10. Fourier-Transform Infrared Spectroscopy

Fourier transform infrared spectroscopy (FTIR) studies were performed using a Spectrum 100 FTIR spectrometer (PerkinElmer, Waltham, MA, USA), and spectra were collected in the range of 400 cm ^−1^ to 650 cm ^−1^ with a total of 16 scans and a resolution of 2 cm^−1^, unless otherwise stated. Background scans were performed for all experiments and each sample was analysed in duplicate to check the reproducibility of the spectra.

### 2.11. E-Tongue Taste-Assessment

The TS-5000Z (Insent Inc., Atsugi-shi, Japan) was equipped a BT0 negatively charged lipid taste sensors and a corresponding reference electrode (New Food Innovation Ltd., Nottingham, UK).

The reference solution was prepared by dissolving 30 mM potassium chloride and 0.3 mM tartaric acid in distilled water. The negatively charged membrane washing solution was prepared by diluting absolute ethanol to 30% (*v/v*) with distilled water followed by the addition of 100 mM hydrochloric acid. A sensor check was conducted routinely before each measurement to ensure that the sensors were working within the correct mV range.

Taste sensor output is obtained by measuring the difference in electric potential between the taste sensor and the reference electrode. Dose–response curves for CPM and quinine HCl dihydrate were generated by testing those drugs at concentration ranging between 0.01 mM and 10 mM, corresponding to concentrations equivalent to intervals on the logarithmic scale.

Each measurement cycle consisted of the following elements:

1. Measurement of reference potential (*Vr*) in reference solution;
2. Measurement of electric potential (*Vs*) in sample (initial taste);
3. Lightly washing of sensors in reference solution;
4. Measurement of electric potential (*V_r1_*) in reference solution again (aftertaste or CPA);
5. Refreshing of sensors in alcohol solution to give them a complete wash before the measurement of the next sample.

*Vs − Vr = initial taste*

*V_r1_ − Vr = aftertaste*

#### 2.11.1. Bitterness Threshold

The bitterness threshold of CPM was calculated as a comparison to quinine HCl dihydrate, a commonly used bitter standard drug with known bitterness and aversiveness levels in humans. In human taste panels, bitterness thresholds are determined by selecting the concentration of the drug that produces half of the maximal rating (100), known as the EC50 [26]. For the E-tongue, bitterness thresholds are deduced by using the human EC50 for quinine HCl dihydrate, and finding the corresponding mean sensor response at that value [26].

Sensor output is proportional to the logarithmic concentration of a test substance, which is based on the Nernst equation:

$$U=U^{0}+\frac{RT}{zF}\;\ln a_{i}$$

where *U* = electrode potential; *U*^0^ = standard electrode potential; *R* = universal gas constant; *T* = temperature (K); *z* = ionic valence of the substance; *F* = Faraday constant; $a_{i}$ = activity of the substance.

$$a_{i}=f_{i}c_{i}\;$$

where $c_{i}$ = concentration of the substance$;\;f_{i}$= activity coefficient of the substance [27].

Therefore, sample concentrations equivalent to intervals on the logarithmic scale were prepared (0.01 mM up to 10 mM).

#### 2.11.2. Sample Preparation of the Fibres

Taste extracted liquids were used for biosensor assessment of the electrospun fibre mats, and materials that are insoluble in the media’s pH, such as KCT and E-EPO. The fibres were added to 100 mL of 10 mM potassium chloride solution, as a supporting electrolyte, at 37 °C and gently stirred for 1 min. The mixture was then filtered through 0.22 µm filters (Merck-Millipore, Cork, Ireland), removing any suspended particles.

#### 2.11.3. Data Analysis

All measurements were repeated four times. The data from the first run were discarded to allow for the conditioning of sensors. In this study, a solution of 10 mM potassium chloride was used as a control sample, and the corresponding sensor responses were subtracted from the sensor responses of the samples. Hence, all data produced are a mean of three measurements and represent relative sensor responses. Multivariate Principal Component Analysis (PCA) was performed on the data collected. This aided visualization of the high number of data points on a two-dimensional map. Differences between samples were assessed by determining the Euclidean distances which were calculated from the cluster center [28]. All data analysis was carried out using OriginPro 9.4 (Origin Lab, Northampton, MA, USA).

### 2.12. In-Vitro Dissolution

A Sciquip mini shaker (SciQuip, Wem, UK) was used to investigate the release of CPM in a pH 1.2 media to mimic the stomach conditions. 0.1 N HCl was used as the dissolution media. One-millilitre aliquots were removed and replaced with fresh media at the following time intervals: (1, 5, 10, 15, 20, 30, 45, 60, 90, and 120) minutes. Removal and replacement of fluid was taken from the same position on all occasions. A media volume of 15 mL was used. Samples were placed in glass vials and the mesh cover dropped over them at the beginning of the time. Drug amount released was measured using UV spectrophotometry at wavelength of 262 nm. Fibre samples measured 12.30 mg ± 0.7 mg and had a theoretical drug-load of 4.76%, 7.6%, or 18.9% (*w/w*). Equation of calibration curve in release media: y = 0.0197x + 0.021, R^2^ = 0.993. A 1 mL quartz cuvette was used, and all experiments were performed in triplicate. Data was collated and analysed using OriginPro 9.4 (Origin Lab, Northampton, MA, USA).

### 2.13. Film Thickness and Folding Endurance

3 cm × 2 cm films were cut. Their thickness were measured using a stainless steel digital calliper D03196 (DuraTool, Taichung, Taiwan) [29]. The calliper had a resolution of 0.01 mm and repeatability of 0.1 mm. Three measurements were taken and an average was calculated.

Folding endurance is defined as the number of times a film can be folded without breaking or visibly cracking. Folding endurance was determined by repeatedly folding the films in the same place. After 30 folds the experiment was stopped and films were determined to be flexible. For each formulation, three samples were examined. This method has been used repeatedly by other researchers [30,31,32].

## 3. Results and Discussion

### 3.1. KCT Electrospinning

**Figure 2:**
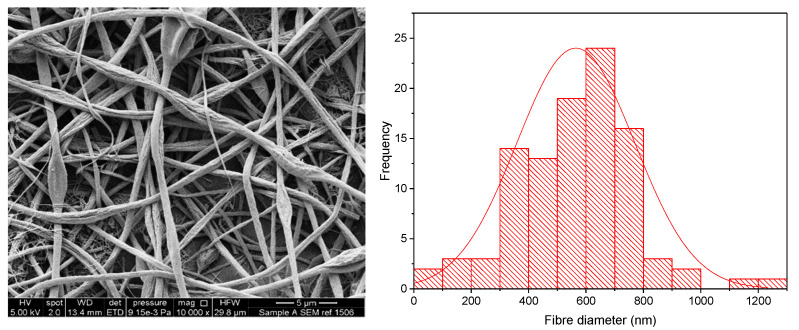
SEM of Electrospun KCT fibres in addition to a fibre diameter distribution histogram.

**Figure 3:**
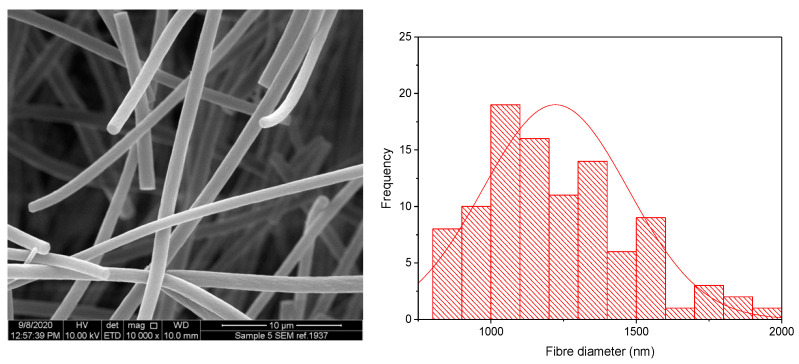
SEM of drug-loaded Electrospun KCT fibres in addition to a fibre diameter distribution histogram.

The KCT polymer solution was successfully electrospun alone at an applied voltage of 20 kV, flow rate of 1 mL/h and gap distance of 17.5 cm. Figure 2 shows KCT fibres using SEM imaging, with a histogram showing the fibre diameter distribution. The mean fibre diameter was measured as 565 nm ± 206 nm, which is within the desired nano-range. It can be seen from the image that the fibres were not perfectly bead-free, with some spindle formation. As this is planned to be used as part of a co-axial electrospinning process, there is leeway for the (core) solution to be less electrospinnable than the second (shell) solution to be used in the process [16]. The drug-loaded fibre had a mean fibre diameter of 1222 nm ± 250 nm and was electrospun at an applied voltage of 8 kV, flow rate of 0.5 mL/h and gap distance of 17.5 cm. An image of the fibres and the histogram fibre diameter distribution is shown in Figure 3. It can be seen that the fibres had overall a narrow fibre diameter distribution and generally had a smooth appearance with no apparent beading. There are no reports in the literature of Kollicoat^®^ Smartseal being electrospun before, hence these data may be of use for future activities in this regard.

### 3.2. Co-Axial Electrospinning

For encapsulating active pharmaceutical ingredients in fibres, core-sheath structures are preferred [33]. This can be achieved using electrospinning or other manufacturing methods. The core can be designed and manufactured to give each fibre desired mechanical properties. The active ingredients can be simultaneously contained in the sheath of the fibres, typically a thin layer compared to the core. This allows the fibrous structures to be more effective in that the all-important functionality is concentrated on the sheath, and thereby delivery is more efficient and effective.

Optimum electrospinning parameters for E-EPO were taken from Abdelhakim et al. [11]. In combination with KCT, the two polymers were alternated between core and shell positions and co-axially electrospun together. The reason they were alternated was to find the optimum conditions for bead-free fibres as well as a fully taste-masked formulation for the bitter drug CPM. Table 1 shows the composition of the different co-axial solutions spun and also contains the drug-loading efficiency and the mean nanofibre diameter.

It can be seen that the two polymers were alternated (systems 2 and 5) as well as repeated (systems 3 and 4) in the core and shell structure. System 1 is a placebo structure that contained E-EPO in the shell and KCT in the core. This arrangement was chosen because E-EPO solution is more electrospinnable than KCT solution hence was placed in the shell solution, believed to be the dominant component for the co-axial structural integrity [34].

All combinations yielded fibres in the nano-range. The placebo fibres had the largest average fibre diameter, likely due to the lack of drug presence and therefore low charge density [35]. The drug-loading efficiency of the co-axial 4 and 5 fibres had a large distribution as indicated by the SD, and this is likely due to intermittent needle clogging and hence uneven distribution of drug particles in some areas of the fibre mat [36]. The system with the narrowest drug-loading distribution was formulation 3, which consisted of only E-EPO. This can be explained by the fact that E-EPO has been previously extensively optimised to be electrospun [11], therefore the jet stream is consistent with minimal clogging or interruption, leading to uniform fibre formation.

**Figure 4:**
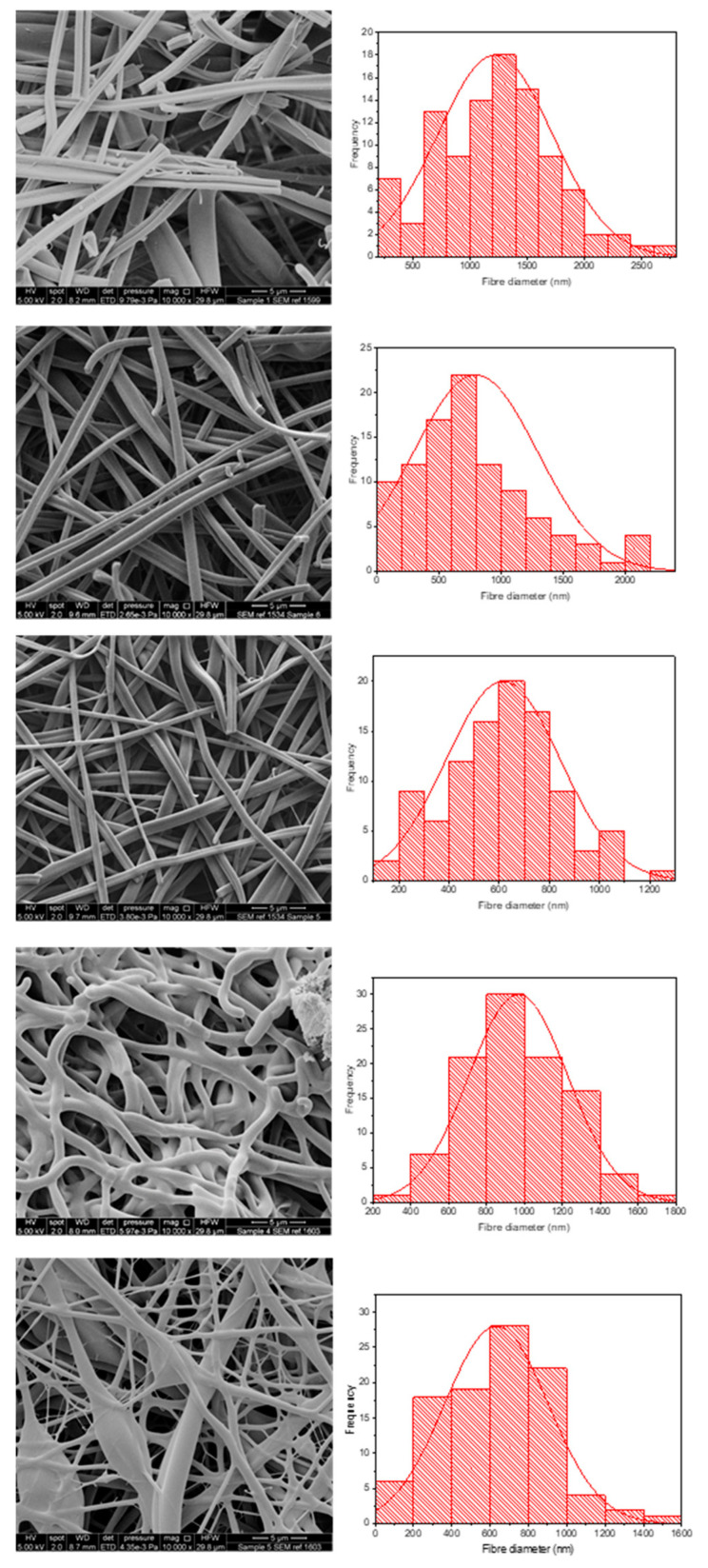
SEM images of the five formulations tested as listed in Table 1. The histograms represent distribution of fibre diameters. The images from top to bottom represent systems 1 to 5. Sample 1 is a placebo formulation whilst samples 2 to 5 contained CPM in the core.

Figure 4 shows the morphology and fibre diameter distribution of the five different spun formulations. It can be seen that systems 1 to 3 generally have a smooth structure, whereas systems 4 and 5 are more spindled. This is likely due to the fact that KCT is in the shell portion of the co-axial structure in those formulations, and due to its low spinnability compared to E-EPO, the fibres are less smooth. Fibres 1 and 2 contain both KCT and E-EPO, with E-EPO being in the shell. As previously mentioned, the needle tip did get clogged and, during cleaning, breakages could occur in the fibres, as seen on the SEM images. System 1 is placebo, whereas system 2 is drug-loaded. This is reflected in the fibre diameter, as the drug-loaded system is expected to have a higher charge density, therefore leading to thinner fibre formation [37].

Systems 2 and 3 are fairly similar because E-EPO is in the shell of both layers; nonetheless, system 3 had a smaller average fibre diameter due to the improved jet elongation and stretching [38]. System 4 had undulant fibres that were not as smooth as E-EPO alone, reflecting the fact that KCT does not spin well on its own; this webbed appearance may occur due to there being an insufficient time for solvent evaporation given the high-water content. System 5 also had KCT in the shell and is therefore less smooth compared to the systems with E-EPO in the shell; however, is still smoother in appearance than 4, possibly due to the presence of E-EPO in the core acting as a template for the shell to solidify around. System 5 had a significantly smaller diameter than the rest of the systems due to the elongation of the fibres between the formed beading and spindles, reflecting the observation that the core solution is spinnable but the shell is less so [39].

**Figure 5:**
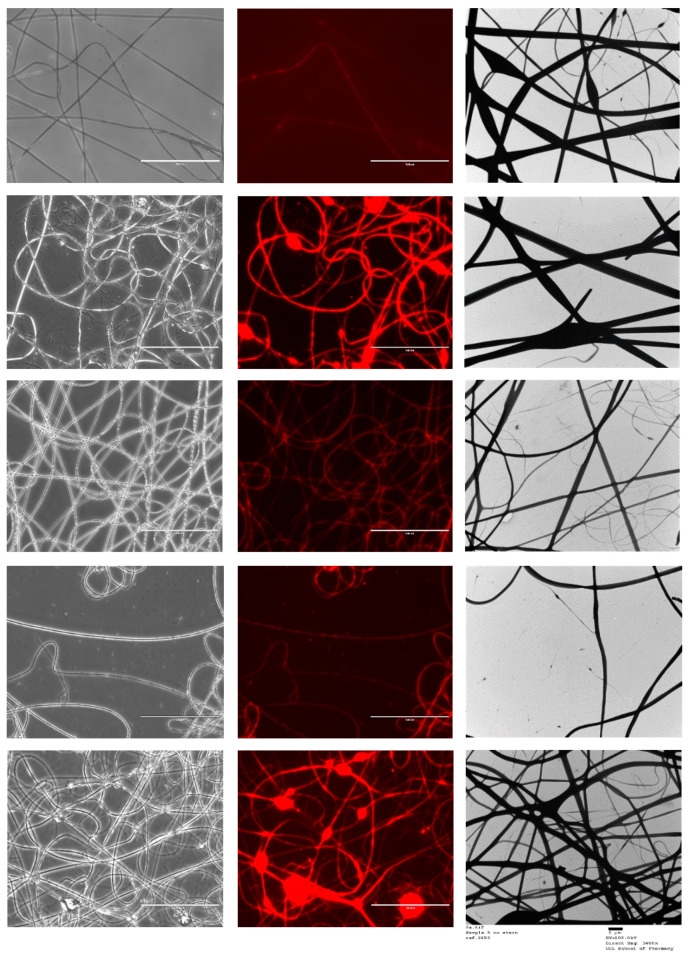
FL microscopy images of the five co-axial formulations. Scalebar represents 100 µm. The images from top to bottom represent samples 1 to 5. TEM images are also represented; scalebar for the TEM images represents 2 µm. Sample 1 is a placebo formulation whilst samples 2 to 5 contained CPM in the core.

Figure 5 shows the FL microscopy images of the samples in both black and white and with the Texas Red light filter, which illuminates the Rhodamine B that was loaded into the core of all the formulations tested. Both the optical microscopy images and the fluorescent images have an identical appearance, indicating that all the nanofibres represented were successfully loaded with the drug-loaded core solution that contained rhodamine. As the fluorescent image shows homogenous distribution of the dye, it can be assumed that the drug has been loaded homogenously in the nanofibres [40]. TEM images are also shown in Figure 5.

### 3.3. Solid State Characterisation

#### 3.3.1. XRD

**Figure 6:**
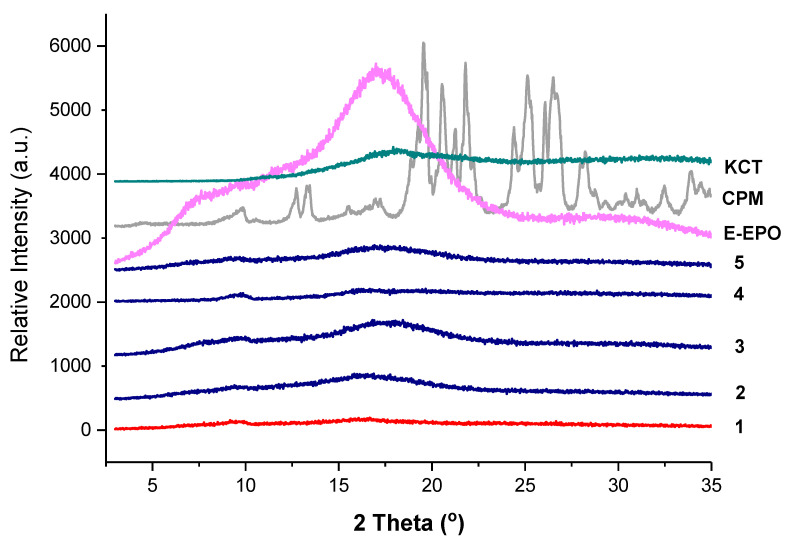
XRD diffraction patterns of pure CPM, E-EPO, KCT and the five co-axial systems electrospun. The formulations labelled 1 to 5 compositions are as detailed in Table 1.

Figure 6 shows the XRD diffraction pattern of all the co-axial systems and the raw drug and polymers. CPM shows a crystalline structure as identified by the clear diffraction peaks. KCT and E-EPO show amorphous structures as shown by the halos. The electrospun fibres clearly lack the distinctive diffractions peaks observed in the crystalline CPM pattern, indicating the formation of an amorphous system in which the drug is molecularly dispersed. The placebo fibre shows a similar diffraction pattern to the drug-loaded diffractograms, confirming this finding.

#### 3.3.2. FTIR

**Figure 7:**
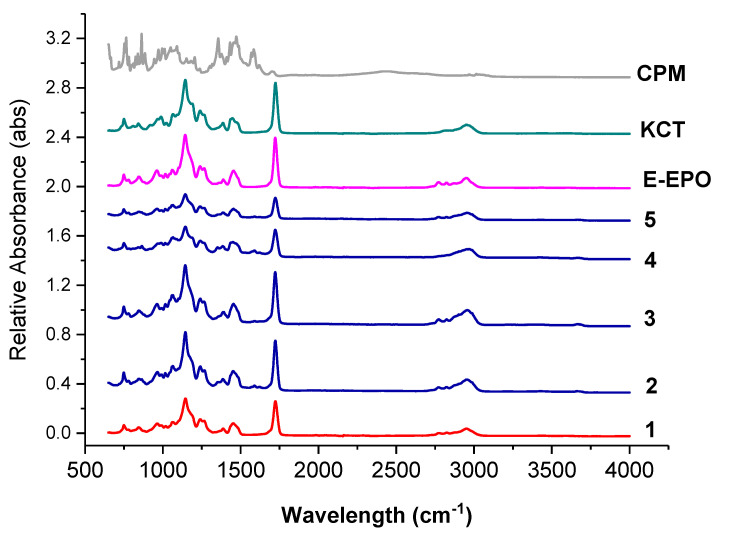
FTIR spectra of pure CPM, raw E-EPO, KCT, and the five co-axial systems electrospun. The formulations labelled 1 to 5 compositions are as detailed in Table 1.

Figure 7 shows the FTIR spectra of the raw ingredients and the electrospun co-axial systems. Methacrylate polymers have characteristic peaks at around 1730 cm^−1^, due to the presence of C = O groups in their molecules. 

These peaks are observed in both KCT and E-EPO spectra. The peak observed in both polymers around 1210 cm^−1^ is characteristic of the C−O bond, also very abundant in both these polymer types. Both those prominent peaks are present in all the co-axially spun fibres (systems 2 to 5), as well as the placebo system, number 1. The spectra of the polymers and fibres are very similar which indicates that no strong bonds were formed between the polymers and the drug CPM, further validating the XRD data that an amorphous solid dispersion was formed. The drug CPM shows characteristic peaks at: 850 cm^−1^ responsible for the C-Cl stretching; 1700 cm^−1^ indicative of C=O stretching; and 1355 cm^−1^ which is indicative of an aromatic amine [41]. These peaks do not seem to present anymore in the fibres which indicates that the functional groups within the drug could have formed hydrogen bonds with the methacrylate polymers. These interactions give an indication of compatibility and therefore stability of the drug in the co-polymer matrix [34]. These results indicate that CPM was amorphously dispersed in the polymeric carriers, which was also validated in the XRD data.

#### 3.3.3. DSC

**Figure 8:**
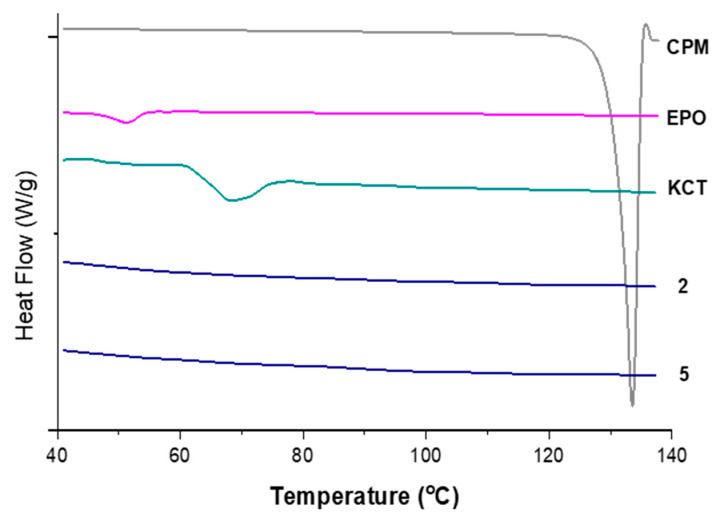
DSC thermograms of pure CPM, raw E-EPO, KCT, and co-axial systems 2 and 5, which contains both polymers as well as the drug. Exo up.

Figure 8 shows the DSC thermograms of pure CPM, the polymers E-EPO and KCT, and co-axial systems 2 and 5. These two were shown here as they contain both polymers alternating between core and shell, and both of them contain the drug CPM in the core. The melting point of CPM, shown by a sharp endothermic peak, is 133.24 °C; this is in close agreement with the literature values of 130–135 °C [42]. E-EPO’s glass transition temperature was recorded as 50.9 °C, consistent with literature findings [43], whilst KCT’s glass transition temperature was measured as 68.4 °C with a previously reported value of 68 °C [44].

**Figure 9:**
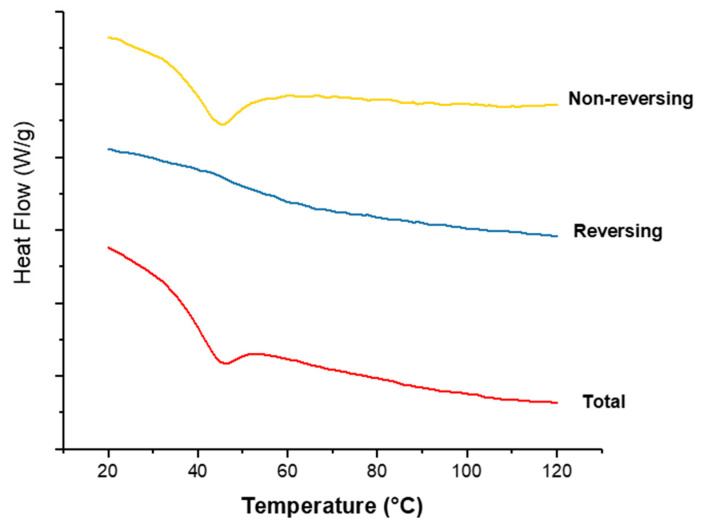
MTDSC thermogram of co-axial system 2 which contained KCT in the core with CPM, and E-EPO in the shell. Exo up.

Figure 9 shows the thermograms of co-axial systems 2. This system was shown here as it contains both polymers alternating between core and shell, and contains the drug CPM in the core. From Figure 8, CPM’s melting point is 133.24 °C; E-EPO’s glass transition temperature is 50.9 °C, whilst KCT’s glass transition temperature was measured around 68 °C. The total heat flow as well as the reversing and non-reversing components are displayed. MTDSC allowed the separation of the glass transition which appears in the reversing heat flow signal, where the non-reversing signal shows the relaxation endotherm [45]. The addition of the drug lowered the *T*_g_ of the system to approximately 44 °C, due to a plasticising effect [46]. This value indicates likely stability at room temperature storage as it lies 10 °C to 20 °C above the storage temperature [47].

### 3.4. Dissolution Study

**Figure 10:**
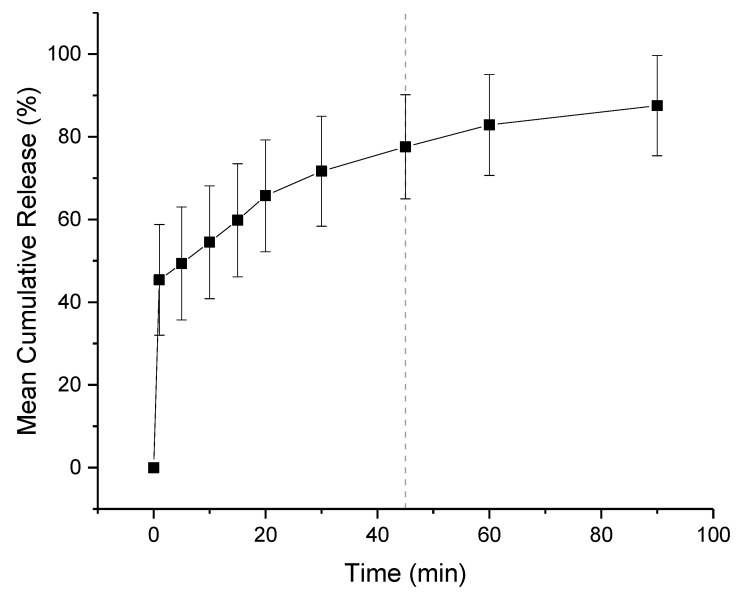
Dissolution profile in pH 1.2 co-axial formulation of KCT and CPM in the core, and E-EPO in the shell. The dashed line represents 45 min.

The in-vitro release profiles of co-axial formulation 2 is shown in Figure 10. The dissolution experiment was performed in a buffer solution of pH 1.2, mimicking fasted gastric conditions. Children over 2 have a similar gastric pH to adults, which is generally between 1.0 and 2.5. Both KCT and E-EPO are pH dependent polymers that are insoluble over pH 5.0 therefore release in pH 6.8 buffer was not performed. It can be seen that the co-axial electrospun film releases rapidly owing to its high surface area to volume ratio, and therefore a high release rate is expected. It can be seen that the film released approximately 75% of the drug amount at 45 min, which is in line with an immediate release formulations’ guideline of releasing 70% of the drug in that timeframe (Ph. Eur. 5.17.1) [48]. The formulation is expected to release fully in the stomach, where no adverse effect due to the taste-masking on absorption is anticipated.

### 3.5. Film Thickness, pH, and Folding Endurance

The thickness of the films is one of the main acceptability parameters for patients. The thickness of a 3 cm × 2 cm co-axial 2 film was taken, and the average measurement was found to be 100 µm ± 20 µm. These thickness levels are in line with literature recommendations [49,50,51] although these values can easily be altered by increasing the time of electrospinning and therefore the amount fabricated.

In addition to thickness of films, folding endurance is a very important parameter for the formulation to effectively be taken by the patient. The films were folded in the same position, and it was found that after bending them 30 times they did not break, which is considered high endurance [30,32]. The fact that the films have high folding endurance indicates that they will be well handled and easily ingestible.

The pH of the films when placed in distilled water was 6.63 ± 0.28, which is approximately neutral pH and within consistent values of saliva’s natural pH levels. This indicated that the formulation is not expected to cause any irritation due to its pH levels [29,52].

### 3.6. E-Tongue Taste-Assessment

#### 3.6.1. Dose–Response Curve

**Figure 11:**
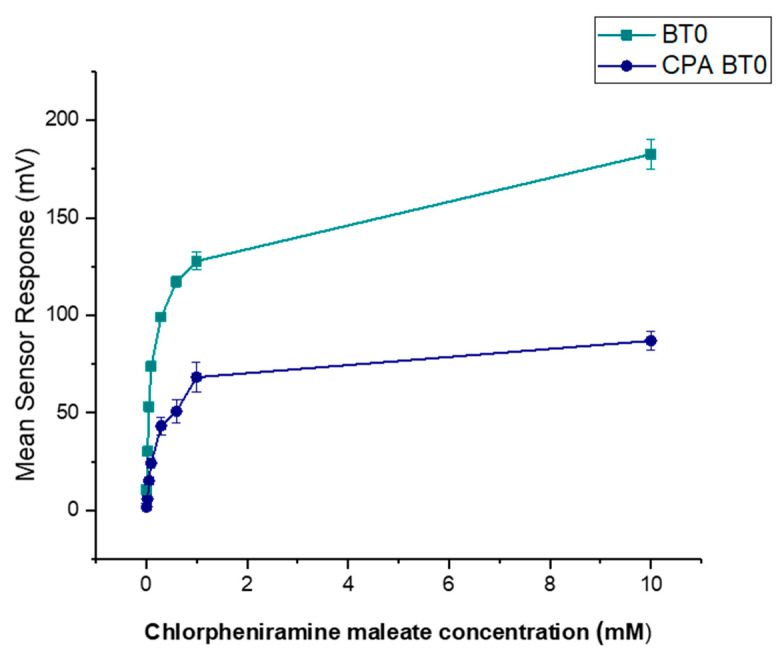
Dose–response curve representing initial taste and aftertaste (CPA) for CPM as detected by BT0 sensor.

It is known that CPM is detectable by the E-tongue’s basic bitterness (AN0 and AC0), acidic bitterness (C00) and astringency (AE1) sensors as verified by our previous study [11]. The E-tongue utilises sensors of various qualities to detect a taste of a substance relative to a reference point. In this case, basic bitterness sensors were used and compared the taste of the various formulations to the raw unformulated drug. Bitterness is associated with adsorption of molecules on the surface of the sensors. Consequently, aftertaste or ‘Change of membrane Potential caused by Adsorption’ (CPA) can be a reliable measure of those tastes. If more than ± 5 mV is recorded by the sensors, this implies that the drug displays this taste quality to some degree. BT0 is the newest basic bitterness sensor with improved selectivity and sensitivity [53] and therefore data from it was used for comparisons. Figure 11 shows BT0′s response as initial taste and aftertaste output (CPA) to raw CPM at concentrations ranging between 0.01 to 10 mM, to ensure detectability. It can be seen that CPM is detected by this sensor and has a perceived basic bitter taste profile. The aftertaste results indicate that this bitterness persists through after light washing, and therefore taste-masking is required. CPM was previously assessed using AC0 and AN0 sensors, both also responsible for detecting basic bitterness taste qualities. AC0, AN0, and BT0 all possess different plasticisers that allow them to have different affinities and therefore sensitivities to differing bitter molecules. Even though the measurements are very similar to that of AN0′s, BT0 has been shown the be more selective [54] and therefore will be used to calculate the bitterness threshold of CPM.

**Figure 12:**
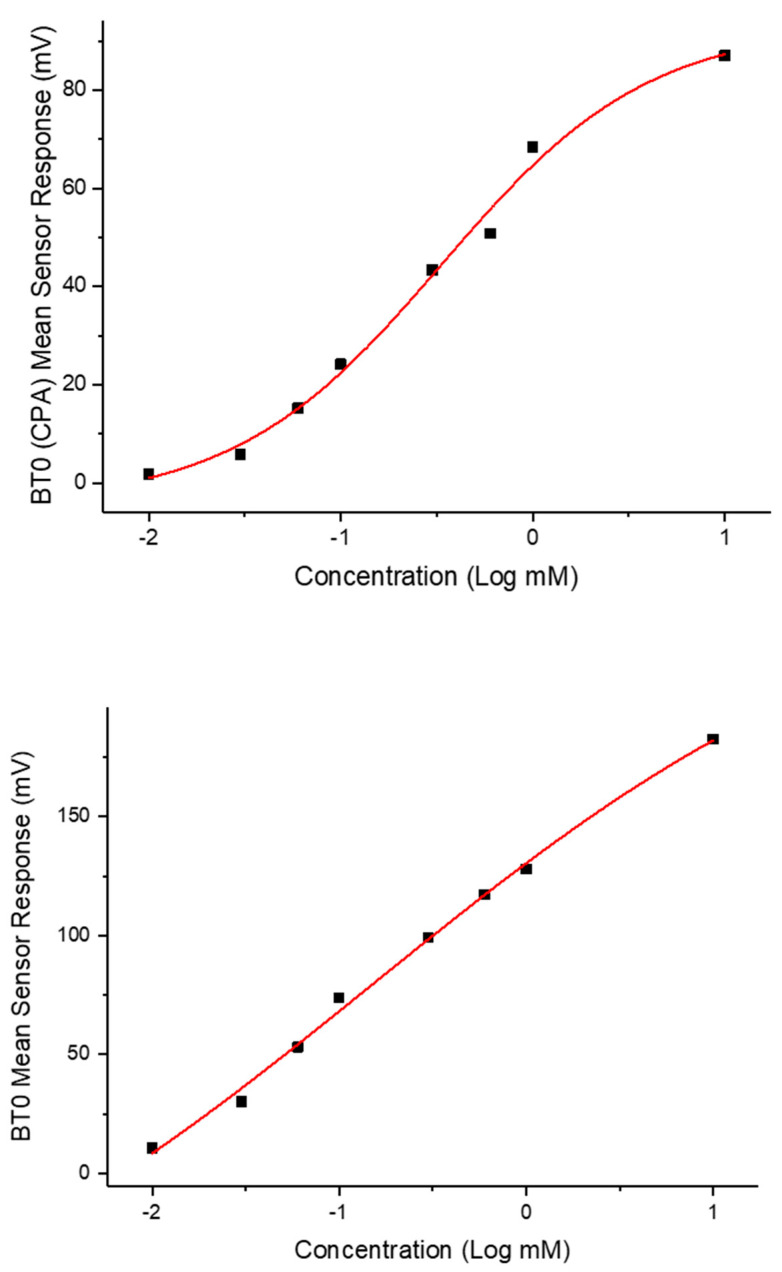
Non-linear Boltzmann fitting of the initial taste (BT0 sensor) and aftertaste (BT0 CPA sensor) of chlorpheniramine maleate.

To ensure BT0 was an appropriate sensor for further analysis and comparison with standard bitter drugs, a non-linear Boltzmann fitting was completed on the data-set for both initial taste and aftertaste. A Boltzmann fitting produces a sigmoidal curve which is indicative of an effect plateauing off when reaching a measured maximum [25]. The effect of taste response is not linear but instead logarithmic as explained by the Weber–Fechner law that states that the relationship between a stimulus and the corresponding perceived intensity is logarithmic [26,27]. As shown in Figure 12, the initial taste represents a sigmoidal curve and thus successful Boltzmann fitting, indicating the use of BT0′s initial taste curve for further analysis is appropriate. The fitting produced an R^2^ = 0.99 for initial taste and R^2^ = 0.98 for aftertaste. 

#### 3.6.2. Bitterness Threshold

**Figure 13:**
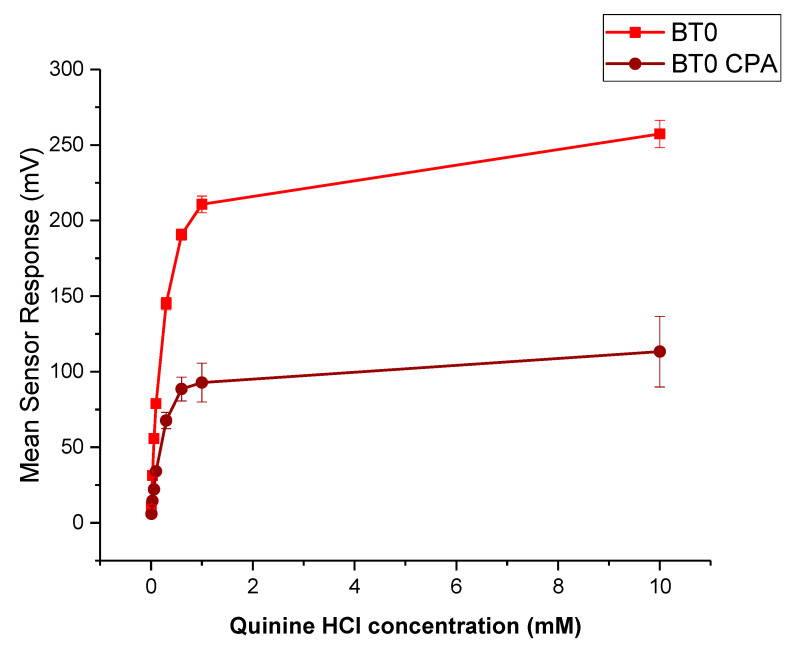
Dose–response curve representing initial taste and aftertaste (CPA) for quinine HCl dihydrate as detected by BT0 sensor.

Quinine HCl dihydrate, an anti-malarial drug, is a commonly used bitter drug standard in taste-assessment studies [55]. The dose–response curve using the BT0 sensor is shown in Figure 13. The drug is well detected by the E-tongue for both initial taste and aftertaste measurements, therefore will be used to determine the bitterness threshold for CPM.

**Figure 14:**
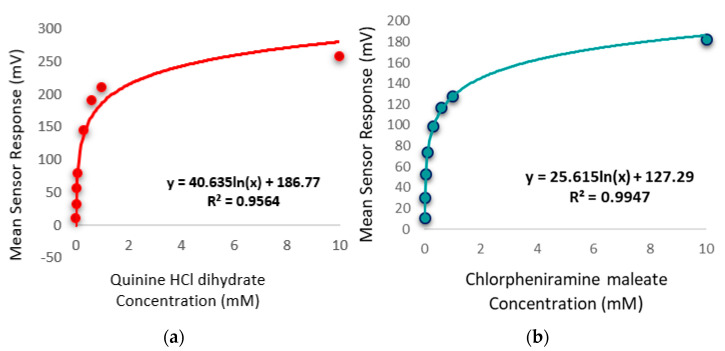
(**a**) Quinine HCl dihydrate response shown as a logarithmic trend-line as determined by BT0 sensor. (**b**) Chlorpheniramine maleate response shown as a logarithmic trend-line as determined by BT0 sensor.

The bitterness threshold for quinine HCl dihydrate from a human sensory test was determined to be 0.26 mM [56] which equates to an E-tongue BT0 sensor output of 132 mV when fitted on a logarithmic trend-line using the equation in Figure 14a.

To estimate the bitterness threshold of CPM, this value was used and substituted into the corresponding logarithmic equation to generate a mean drug concentration that matches quinine HCl dihydrate’s known bitter mean sensor response (132 mV). The logarithmic fitting of the data is shown in Figure 14b. Using data from the BT0 sensor, the bitterness threshold of CPM was calculated to be 1.2 mM or 0.47 mg/mL, meaning any concentration above this point is expected to exert a bitterness response by the person tasting it, and therefore requires taste-masking. Since the clinical CPM dose is between 1 mg and 4 mg [57], and the average human secretes 1 mL of saliva per minute [58], this threshold is below the clinical dose and therefore taste-masking of this drug is essential. In the literature the human bitterness threshold of CPM was reported to be 0.506 mg/mL or approximately 1.3 mM [59]. This means that the E-tongue is reporting a slightly lower value, which may indicate higher sensitivity compared to in-vivo findings. This can be expected as humans vary in sensitivity levels, with a proportion of people classified as non-tasters, thereby potentially skewing aversiveness results [60]. This subjectivity limitation is therefore eliminated when using a biosensor. Nonetheless, the values are in close agreement, indicating good E-tongue to human correlation.

#### 3.6.3. Formulations Taste-Assessment

**Figure 15:**
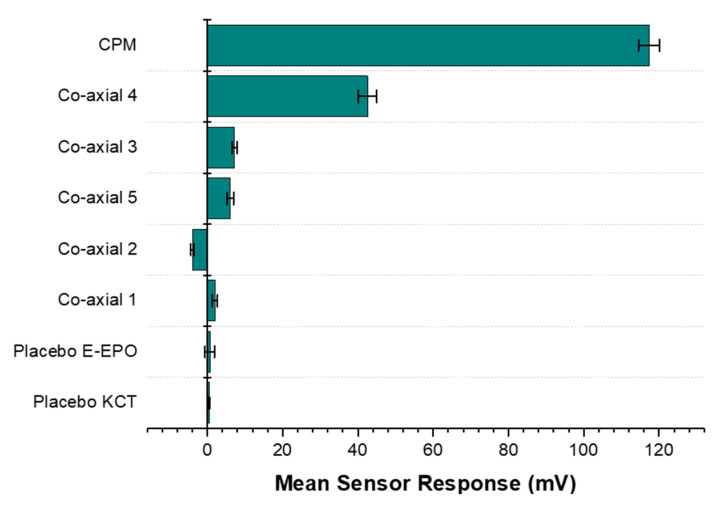
A bar chart representing the E-tongue’s response to the various formulations also tested placebo fibres. The sensor used for this assessment was BT0, the newest generation basic bitterness sensor.

Results from the BT0 sensor responsible for detecting bitterness of basic salts, are shown in Figure 15. It can be seen from the bar chart that the raw unformulated drug, CPM, gave the highest sensor response and is at the top of the chart indicating the highest bitterness. Co-axial system 4 appeared to exhibit the weakest taste-masking. This is likely due to the fact that it did not contain any E-EPO, which was present in a much higher concentration thereby providing better protection of the enclosed API. Similarly, the next system was co-axial 3, which also contained one polymer in both the core and the shell, E-EPO. It did, however, exhibit much higher taste-masking than co-axial system 4. This is likely due to its more superior electrospinnability and therefore stability of the fibres formed. Also, E-EPO was electrospun at 35% (*w/v*) whereas KCT was electrospun at 7.5% (*w/v*). The higher concentration of E-EPO provided a thicker structure that gave rise to better formed fibres and superior taste-masking.

Co-axial system 5 contained E-EPO in the core and KCT in the shell. The combination of both polymers seems to be better than either polymer alone. However, looking at their SEM images, the fibres formed were spindled due to low electrospinnability of KCT when placed in the shell of the structure.

Any mean sensor response between −5 mV and 5 mV is deemed undetectable by the E-tongue’s sensors. Co-axial system 2 was deemed undetectable by the E-tongue, as the sensor was stabilising in the negative range and the measurement was within –5 mV to 5 mV, showing effective taste-masking. This is likely due to the fact that E-EPO was in the shell, and because it was used at a higher concentration than KCT, it is hypothesised that a thicker more protective shell was formed, causing the superior taste-masking. The placebo fibres were undetectable, as expected.

Principal component analysis was used to reduce data from the initial taste and aftertaste of BT0 and AN0 sensors into a two dimensional output [61]. Principal component 1 was responsible for 71.97% of the variance in the data and it was determined by data outputs from the BT0 sensor, indicating it had most influence on the results. The 2nd principal component was determined by data from the AN0 sensor and was responsible for 27.83% of the variance in the data. Both principal components therefore account for 99.82% of the variance of the data demonstrating that these results are credible and not merely due to chance.

Table 2 shows the Euclidean distance calculated for the various formulations from the bitterest point, the raw drug. The further away the distance, the more taste-masked a formulation is. The most taste-masked formulation is co-axial formulation 2, which contained KCT in the core with the drug, and E-EPO in the shell. Perhaps this is a somewhat expected outcome as E-EPO electrospins well, and therefore when loaded in the shell of the formulation an intact and taste-masked fibre mat is formed.

Co-axial systems 3 and 5 were taste-masked similarly as shown by the comparable Euclidean distance values. Co-axial system 4 was the closest to the raw drug, indicating the least taste-masking and therefore the lowest Euclidean distance.

## 4. Conclusions

KCT Smartseal was successfully electrospun for the first time as far as the authors are aware. The conditions were optimized to produce smooth bead-free fibres. Using the optimized conditions, KCT and E-EPO were both used in conjunction to co-axially electrospin CPM for taste-masking. Co-axially electrospun fibres show improved taste-masking compared to raw drug. In addition, combining both polymers in one co-axial system improved taste-masking compared to either polymer alone. It was concluded that placing E-EPO in the shell solution drives the electrospinning process in a much more efficient manner compared to KCT, as in co-axial system 2. However, even though co-axial system 2 forms taste-masked nanofibres that are confirmed to be an amorphous solid dispersion, needle clogging is still problematic and will require further optimization.

The fibre release study showed that drug was released in acidic media within the pharmacopeial (Ph. Eur. 5.17.1) requirements of 70% release within 45 min for a formulation to be considered immediate release, indicating that on reaching the stomach the release will allow suitable drug absorption within the gastrointestinal tract. The corresponding spun film thickness (using formulation 2) was comparable to literature values, while the folding endurance of the films was high, showing high potential acceptability of the films when handled by the patient. In addition, when the films were dispersed in distilled water, the pH measured was consistent with natural saliva pH, indicating low mucosal irritability as a result of the ingestion of this oral film.

The E-tongue was used to measure the taste of CPM’s bitterness as well as the associated formulations. The bitterness threshold of CPM was calculated which was used to ascertain whether the drug released from the formulation surpasses that value thereby eliciting aversiveness.

Overall, it was shown that co-axial electrospinning using dual taste-masking polymers is a promising technique to manufacture taste-masked formulations that have the potential to be further formulated into age-appropriate dosage forms such as oral films.

## Figures and Tables

**Table 1:** The formulations tested had KCT and E-EPO alternating between the core and shell solutions. CPM was always added at 3.5% (*w/v*) in the core apart from system 1, a placebo. The table shows the drug loading efficiency and mean diameter of the nanofibres. DL= drug loading.

Co-Axial Sample	Core	Shell	Theoretical DL (% *w/w*)	Actual DL (% *w/w*)	DL Efficiency (%)	Mean ± SD Diameter (nm)
1	KCT	E-EPO	N/A	N/A	Placebo	1220 ± 501
2	KCT	E-EPO	7.6	7.4 ± 0.4	97.5 ± 5.3	795 ± 505
3	E-EPO	E-EPO	4.8	5.3 ± 0.2	111.3 ± 4.2	616 ± 228
4	KCT	KCT	18.9	17.8 ± 3.6	94.2 ± 19.0	967 ± 262
5	E-EPO	KCT	7.6	7.0 ± 1.6	92.1 ± 21.1	633 ± 271

**Table 2:** Euclidean distance of the drug-loaded co-axial formulations from 20 mg/100 mL or 0.5 mM CPM.

Formulation	Euclidean Distance
Co-axial 4	4.43
Co-axial 5	7.51
Co-axial 3	7.63
Co-axial 2	9.40
